# Tenecteplase compared to alteplase in real-world outcome: A Swedish Stroke Register study

**DOI:** 10.48101/ujms.v129.10459

**Published:** 2024-10-09

**Authors:** Mikael Skärlund, Signild Åsberg, Marie Eriksson, Erik Lundström

**Affiliations:** a Department of Medicine, Nyköping Hospital, Nyköping, Sweden; b Department of Medical Sciences, Neurology, Uppsala University, Uppsala, Sweden; c Department of Statistics, USBE, Umeå University, Umeå, Sweden

**Keywords:** Ischemic stroke, intravenous thrombolysis, tenecteplase, alteplase

## Abstract

**Background:**

Tenecteplase is increasingly used off-label as an alternative to alteplase for ischemic stroke thrombolysis. Our aim was to evaluate the safety of tenecteplase versus alteplase in comprehensive real-world data.

**Methods:**

We compared the outcomes for adult patients with acute ischemic stroke treated with alteplase or tenecteplase, registered in the Swedish Stroke Register between January 1, 2018 and December 31, 2020. The primary outcome was symptomatic intracerebral hemorrhage or death during hospital stay. Secondary outcomes were death within 90 days, modified Rankin Scale at 90 days, and mean door-to-needle time (DNT).

**Results:**

There were no significant differences in age or risk factors between 6,560 patients (45% women, mean age 74) treated with alteplase and 888 patients (43% women, mean age 74) treated with tenecteplase, although tenecteplase was more commonly used in non-university hospitals, hospitals with high use of thrombolysis, and in wake-up strokes. Tenecteplase was not non-inferior compared to alteplase in terms of symptomatic intracerebral hemorrhage or death during hospital stay (13.2% vs. 10.7%, absolute risk difference [95% confidence interval, CI] 2.5% [0.1 to 4.9%], adjusted odds ratio 1.44 [1.07–1.94]). There were no significant differences in functional outcome or death at 90 days, but tenecteplase was associated with decreased DNT (mean difference 9 min).

**Conclusion:**

Tenecteplase was not non-inferior in safety outcome, although associated with decreased DNT. As accumulating randomized controlled studies support the non-inferiority of tenecteplase regarding functional outcome, it is important to keep scrutinizing the safety outcomes.

## Introduction

Thrombolysis treatment of acute ischemic stroke aims to reduce clot burden and restore adequate blood flow to the brain tissue before permanent damage. Fast-administered intravenous fibrinolytic therapy with the tissue plasminogen activator alteplase (ALT) leads to decreased long-term disability, at the cost of higher risk of intracerebral hemorrhage, and has profound support in research and guidelines (1, 2). Tenecteplase (TNK) is a bioengineered tissue plasminogen activator with increased half-life and increased binding to fibrin, that can be administered as a single bolus dose instead of a 1 h infusion (3). In several randomized clinical trials and following meta-analyses, TNK has proven to have non-inferior efficacy and comparable safety as alteplase in ischemic stroke, but with potential for increased early re-canalization and early neurological improvement (4–6). Doses of 0.1, 0.25, and 0.4 mg/kg have been tested, with 0.25 mg/kg (maximum 25 mg/kg) as the most common dosage (7). Data from real-world settings comparing TNK and ALT have not demonstrated any safety concerns, with no difference in symptomatic intracerebral hemorrhage (sICH), and comparable or decreased mortality with TNK. Real-world data also indicate that a switch from ALT to TNK seems to improve recanalization, increase early neurological improvement, decrease door-to-needle time (DNT), and either improve or not alter functional outcome (8–11).

In February 2023, the European Stroke Organisation issued new guidelines supporting the use of TNK 0.25 mg/kg as an alternative to ALT (12). This differs from the previous European Stroke Organisation guidelines and the Swedish National Board of Health and Welfare recommendations that discouraged the use of TNK as a thrombolytic agent in routine care for unselected patients (2, 13). From 2018 to 2020, contrary to the advice by national authorities, 14 Swedish hospitals, including 1 university hospital, decided to switch from ALT to TNK as their standard thrombolytic agent for all eligible ischemic strokes. The decision has been made by local stroke doctors at each hospital considering the existing evidence and mainly with the argument of faster delivery, which by decreased DNT could improve outcome. In 2020, 13% of all ischemic stroke patients in the Swedish Stroke Register were treated with thrombolysis, and of these were 21% given TNK and 79% given ALT (14).

The primary objective of this study was to investigate whether TNK was non-inferior to ALT in safety outcome in a real-world setting, secondary objectives were to investigate differences in DNT and functional independence all using comprehensive Swedish register data.

## Materials and methods

All patients ≥18 years old treated with thrombolysis between January 1, 2018 and December 31, 2020 and registered in the Swedish Stroke Register (Riksstroke) were included in the study. Riksstroke is a nationwide quality register with proven high reliability that records approximately 90% of all cases of acute stroke in Sweden, including all hospitals admitting acute stroke patients in Sweden (14, 15). In 2018, Riksstroke started to register for TNK treatment as one hospital had begun to use TNK routinely. From Riksstroke, data were extracted concerning sex, age, prior need of assistance, past medical history, stroke severity and management during hospital stay including thrombolytic agent used, DNT, thrombectomy, National Institutes of Health Stroke Scale (NIHSS; a score rating neurological stroke symptoms ranging from 0, no symptoms, to 42) (16) on admission, sICH (defined as any intracerebral hemorrhage associated with ≥ 4 points increase in NIHSS) within 36 h, and death during hospital stay. These data are assessed by the local physician and registered locally at each hospital. Three months after stroke, a local coordinator (often a registered nurse) controls for death through public registers and sends a questionnaire to fill in for all living patients or their relatives. From the questionnaire functional outcome using the modified Rankin Scale (mRS, scale ranges from 0 [no neurological deficit] to 6 [death]) (16) was extracted through an earlier validated algorithm differentiating between mRS 0–2, 3, 4, 5 and 6 (17).

We hypothesized that TNK was non-inferior to ALT regarding safety, and our primary outcome was a composite of death during hospital stay and/or sICH. Secondary outcome variables were death during hospital stay or sICH analyzed separately; death within 90 days; DNT, and functional independence 90 days after stroke, defined as mRS 0–2.

Ethical approval for this study was obtained from the Swedish Ethical Review Authority (DNR 2021-01323, Lund 2021-04-06), as well as the Riksstroke board. Registered as an observational study at Uppsala University Hospital registry, number 977075, 12 March 2022. No informed consent is required for quality register-based studies, but registered patients were verbally informed that they could opt out from the registry if they did not wish to participate in studies.

### Statistical analyses

Groups (TNK or ALT) were compared using the chi-square test for categorical variables, t-test for numeric variable and Mann–Whitney U test for ordinal variables, with an alpha level of 0.05. For the primary composite outcome (death in hospital or sICH), we set a non-inferiority margin at 1% absolute risk increase, defining only results with 95% confidence intervals of risk difference better than this as an acceptable non-inferiority. We chose this level for non-inferiority in accordance with earlier studies (4). Absolute risk differences between treatments were given with 95% confidence interval according to Agresti-Caffo. As thrombolysis agent used was entirely based on planned institutional shifts, we did not expect any confounding, and the primary analysis was based on unadjusted differences. To determine whether results in risk differences were subject to controllable bias we conducted a logistic regression analysis for all binary outcomes adjusting for pre-specified factors based on clinical relevance and access in the register. Model 1 included previous factors: age, NIHSS score on arrival, need of daily assistance prior to stroke, treatment year, diabetes, previous stroke, ongoing aspirin treatment, ongoing anticoagulant treatment, wake-up stroke, treated at university center, percentage of all ischemic strokes thrombolysed at receiving center. Model 2 also added factors that may have been influenced by thrombolysis agent: DNT and thrombectomy. Two sensitivity analyses were performed, one excluding patients that were treated with TNK 0.4 mg/kg or unknown dose, as that higher dose lately has been proven inferior (18), and one only including patients before and after the switch of standard drug from ALT to TNK at the same hospitals, in an effort to remove selection bias associated with different centers serving different populations and other differences in stroke care. IBM SPSS version 28 was used for statistical analyses.

## Results

**Figure 1:**
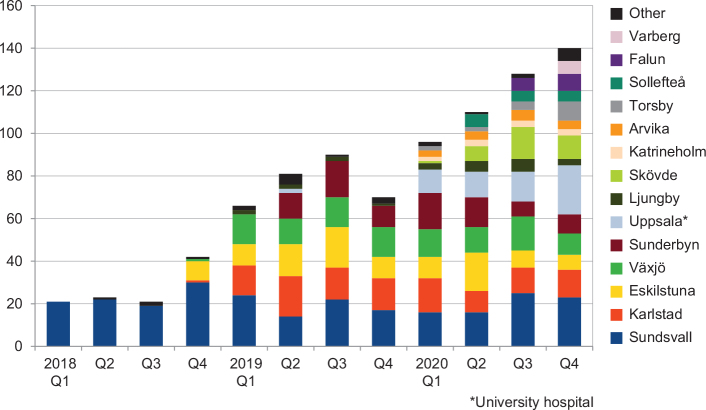
Number of tenectplase treatments per quarter of year in Sweden.

Between January 1, 2018 and December 31, 2020 (3 years), the number of stroke units in Sweden using TNK as standard drug for thrombolysis of ischemic stroke increased from 1 to 14 (Figure 1). During the study period, 888 doses of TNK and 6,560 doses of ALT were registered. Out of the TNK doses, 857 (97%), and 30 (0.5%) of the ALT doses were administered in hospitals which had switched to TNK. One hospital – which contributed with 28% of all TNK patients – switched to TNK before 2018, initially with the dose 0.4 mg/kg, and thereafter gradually switching to 0.25 mg/kg in 2020, while all other hospitals used 0.25 mg/kg, max 25 mg. Of TNK-treated patients, 78% (696/888) were given 0.25 mg/kg, 7% were given 0.4 mg/kg (59/888), and 15% received an unknown dose (133/888). Regarding ALT, all hospitals used 0.9 mg/kg, max 90 mg. The proportion of all stroke thrombolysis with TNK increased from 4% in 2018 (107/2,607) to 21% in 2020 (474/2,277). Six hospitals contributed to 81% of the TNK cases.

The two treatment groups were similar in age and risk factors for stroke. During the study period, only one comprehensive stroke unit (a university hospital with capacity for thrombectomy) used TNK; hence, patients treated with TNK were more often admitted to primary stroke units (non-university hospitals). Moreover, they were more often wake-up strokes and more often treated in hospitals with higher rates of thrombolysis than ALT-treated patients (Table 1).

**Table 1:** Baseline characteristics.

	Valid, *n* (%)	Tenecteplase	Alteplase	*P*
Total, *n*	7,448	888	6,560	
Treated 2018	2,606	107	2,499	
Treated 2019	2,567	307	2,260	
Treated 2020	2,275	474	1,801	
Age in years, mean (Standard Deviation)	7,448 (100)	73.8 (12.3)	73.7 (12.9)	0.90*
Female sex, *n* (%)	7,448 (100)	382 (43.0)	2,955 (45.0)	0.25^†^
Requires assistance in daily life prior to the stroke, *n* (%)	7,312 (98.2)	173 (19.6)	1,135 (17.6)	0.15^†^
Diabetes, *n* (%)	7,427 (99.7)	188 (21.3)	1,317 (20.1)	0.43^†^
Smoker, *n* (%)	6,420 (86.2)	99 (12.9)	772 (13.7)	0.59^†^
- smoker missing, *n* (%)	1,028 (13.8)	123 (13.9)	905 (13.8)	0.96^†^
Hypertension, *n* (%)	7,414 (99.5)	603 (68.5)	4,277 (65.5)	0.07^†^
Atrial fibrillation, *n* (%)	7,374 (99.0)	235 (26.8)	1,631 (25.1)	0.29^†^
Previous stroke, *n* (%)	7,424 (99.7)	171 (19.3)	1,093 (16.7)	0.051^†^
Aspirin, ongoing treatment, *n* (%)	7,422 (99.7)	229 (25.9)	1,765 (27.0)	0.49^†^
Any anticoagulant, ongoing treatment, *n* (%)	7,421 (99.6)	28 (3.2)	204 (3.1)	0.94^†^
Wake-up stroke, *n* (%)	7,286 (97.8)	122 (14.2)	425 (6.6)	**<0.001** ^†^
NIHSS on arrival median (Interquartile range [IQR])	6,758 (90.7)	6 (3–11)	7 (4–14)	0.26^‡^
-NIHSS on arrival missing, *n* (%)	690 (9.3)	104 (11.7)	586 (8.9)	**0.007** ^†^
Percentage of ischemic strokes thrombolysed at receiving hospital, median (IQR)^§^	7,413 (99.5)	21.3% (14.3–23.7%)	14.0% (12.3–16.0%)	**<0.001** ^‡^
Treated at university hospital, *n* (%)	7,413 (99.5)	68 (7.7)	1,814 (27.8)	**<0.001** ^†^
Thrombectomy, *n* (%)	7,426 (99.7)	134 (15.2)	1,143 (17.5)	**0.10** ^†^

* Independent T-test.

† Chi-2 test.

‡ Mann–Whitney U-test.

§ Percentage of all ischemic strokes thrombolysed at the hospital where patient was thrombolysed, according to Riksstroke’s yearly reports (14, 19, 20).

Bold: Significant with *p* < 0.05.

Patients treated with TNK had higher rates of negative results, measured by the combined outcome of death during hospital stay or sICH, 13.2%, compared to 10.7% for those treated with ALT (absolute risk difference of 2.4%). Although this difference was not significant in the two sensitivity analyses: 1) TNK compared with ALT only in the 13 hospitals switching standard drug for thrombolysis during the period (13.1% compared to 12.0%), 2) TNK only in the dose of 0.25 mg/kg compared with ALT (12.8% compared to 10.7%). The unadjusted absolute risk differences and 95% confidence intervals (CI) for the primary composite outcome were above the predefined non-inferiority margin of 1% in all analyses; for the primary analysis in the whole sample: 2.4% (95% CI: 0.1 to 4.9%), only in hospitals switched to TNK: 1.1% (−2.4 to 4.7%), only in TNK in the dose of 0.25 mg/kg, compared to all ALT 2.1% (−0.5 to 4.7%). However, regarding secondary outcomes, there were no significant differences in the rate of sICH (absolute risk difference of TNK vs. ALT 0.6% [−0.8 to 2.3%]) or death during hospital stay (1.8% [−0.2 to 3.9%] when analyzed separately, neither any difference in rate of functional independence (mRS 0–2) after 90 days (1.7% [−2.2 to 5.6%]) nor death within 90 days (1.9% [−0.5 to 4.3%]). Mean DNT was 9 min shorter (11 – 7 min) for those treated with TNK (Table 2). The median and mean survival time for patients deceased in hospital did not differ between TNK and ALT patients (mean: TNK 9.9 days, ALT 9.3 days; median TNK 6 days, ALT 6 days).

**Table 2:** Primary and secondary outcomes.

	Valid, *n* (%)	Tenecteplase	Alteplase	Risk difference % (95% CI*)
**All patients**				
Total, *n*	7,448 (100)	888	6,560	
Death in hospital or sICH†, *n* (%)	7,356 (98.7)	115 (13.2)	696 (10.7)	**2.4 (0.1 to 4.9)**
sICH†, *n* (%)	7,299 (98.0)	43 (5.0)	280 (4.4)	0.6 (−0.8 to 2.3)
Death in hospital, *n* (%)	7,448 (100)	88 (9.9)	533 (8.1)	1.8 (−0.2 to 3.9)
Death within 90 days, *n* (%)	7,448 (100)	125 (14.1)	802 (12.2)	1.9 (−0.5 to 4.3)
modified Rankin Scale (mRS) 0–2 after 90 days, *n* (%)	5,919 (79.5)	375 (52.8)	2,662 (51.1)	1.7 (−2.2 to 5.6)
Missing mRS, *n* (%)	1,529 (20.5)	178 (20.0)	1,351 (20.6)	−0.5 (−3.3 to 2.3)
DNT‡, mean (Standard deviation [SD]), minutes	6,961 (93.5)	34 min (26)	43 min (28)	**−9 min (−11 to −7)**
**Tenecteplase 0.25 mg/kg**				
Total, *n*	7,256 (100)	696	6,560	
Death in hospital or sICH†, *n* (%)	7,164 (98.7)	87 (12.8)	696 (10.7)	2.0 (−0.5 to 4.7)
sICH†, *n* (%)	7,108 (98.0)	36 (5.3)	280 (4.4)	1.0 (−0.7 to 2.9)
Death in hospital, *n* (%)	7,256 (100)	65 (9.3)	533 (8.1)	1.2 (−1.0 to 3.6)
Death within 90 days, *n* (%)	7,256 (100)	95 (13.6)	802 (12.2)	1.4 (−1.2 to 1.4)
mRS 0–2 after 90 days, *n* (%)	5,751 (79.3)	284 (52.4)	2,662 (51.1)	1.3 (−3.1 to 5.7)
Missing mRS, *n* (%)	1,505 (20.7)	154 (22.1)	1,351 (20.6)	1.5 (−1.6 to 4.8)
DNT‡, mean (SD)	6,775 (93.4)	37 min (27)	43 min (28)	**−6 min (−8 to −4)**
**Only centers switching standard drug during period**				
Total, *n*	1,402 (100)	608	794	
Death in hospital or sICH†, *n* (%)	1,375 (98.1)	78 (13.1)	94 (12.0)	1.1 (−2.4 to 4.7)
sICH†, *n* (%)	1,360 (97.0)	34 (5.8)	34 (4.4)	1.4 (−1.0 to 4.7)
Death in hospital, *n* (%)	1,402 (100)	57 (9.4)	76 (9.6)	−0.2 (−3.3 to 2.9)
Death within 90 days, *n* (%)	1,402 (100)	81 (13.3)	95 (12.0)	1.4 (−2.1 to 4.9)
mRS 0–2 after 90 days, *n* (%)	1,118 (79.7)	243 (52.3)	342 (52.7)	0.9 (−5.0 to 6.8)
Missing mRS, *n* (%)	284 (20.3)	139 (22.9)	145 (18.3)	**4.6 (0.3 to 8.9)**
DNT‡, mean (SD), *n* (%)	1,298 (92.6)	37 min (26)	47 min (27)	**9 min (−6 to −12)**

* 95% Confidence Interval of independent samples proportions according to Agresti-Caffo or independent samples T-test accordingly.

† sICH; symptomatic intracerebral hemorrhage defined as intracerebral hemorrhage combined with a deterioration of at least 4 point in NIHSS within 36 h.

‡ DNT; door-to-needle time in minutes, for those suffering stroke during hospital stay (6.1%) door-to-needle = symptom onset-to-needle. Outliers excluded, defined as >75th percentile + 3 x interquartile range or <5 min.

Bold: Significant with *p* < 0.05.

Logistic regression demonstrated an increased odds ratio for the combined primary outcome of death in hospital or sICH with TNK both in the univariate model (1.26 [CI: 1.02–1.56]) and when adjusted for baseline characteristics, DNT and thrombectomy (1.44 [CI: 1.07–1.94]). In the smaller sample, only comparing hospitals before and after the switch from ALT to TNK, 0.25 mg/kg, TNK was associated with a significantly worse outcome only in the most adjusted model, also including DNT and thrombectomy (adjusted OR 1.91 [CI: 1.04–3.51]) (Table 3).

**Table 3:** Logistic regression odds ratios (95% Confidence Interval) for tenecteplas versus alteplase for different outcomes.

	All patients, *N* = 7,448			Only switching hospitals *N* = 1,402		
	Univariate Tenecteplase	Model 1*	Model 2†	Univariate Tenecteplase	Model 1*	Model 2†
Death in hospital or sICH	**1.26** **(1.02–1.56)**	**1.36** **(1.03–1.81)**	**1.44** **(1.07–1.94)**	1.11 (0.80–1.52)	1.54 (0.88–2.68)	**1.91** **(1.04–3.51)**
sICH	1.15 (0.83–1.60)	1.28 (0.85–1.92)	1.31 (0.86–2.01)	1.36 (0.82–2.18)	1.91 (0.85–4.33)	2.13 (0.89–5.10)
Death in hospital	1.24 (0.98–1.58)	1.37 (0.97–1.91)	1.39 (0.98–1.97)	0.98 (0.68–1.40)	1.44 (0.76–2.73)	1.85 (0.91–3.74)
Death within 90 days	1.18 (0.96–1.44)	1.23 (0.92–1.63)	1.20 (0.89–1.62)	1.13 (0.82–1.55)	1.34 (0.76–2.36)	1.57 (0.85–2.90)
mRS 0–2 at 90 days	1.07 (0.92–1.25)	1.04 (0.83–1.31)	1.01 (0.80–1.29)	0.97 (0.76–1.22)	0.98 (0.61–1.58)	0.94 (0.56–1.56)

* Model 1 = Tenecteplase, Age, NIHSS score on arrival, need of daily assistance prior to stroke, treatment year, diabetes, previous stroke, ongoing aspirin treatment, ongoing anticoagulant treatment, wake-up stroke, treated at university center, percentage of all ischemic strokes thrombolysed at receiving center (total missing in model 13.5% for all patients; 14.5% for switching hospitals).

† Model 2 = Model 1 + thrombectomy, door-to-needle time (total missing in model 18.3 and 19.6% respectively).

Bold: Significant with *p* < 0.05.

## Discussion

Our study failed to show TNK non-inferior to ALT in its primary composite outcome (absolute risk difference 2.4% [0.1 to 4.9%] adjusted odds ratio 1.44 [1.07–1.94]). Some of the negative results for TNK in this study can probably be attributed to the usage of TNK in the dose of 0.4 mg/kg. In the recently published randomized trial NOR-TEST 2, this dosage was associated with increased mortality and increased rate of sICH compared to ALT (18), while the EXTEND-IA TNK part 2 trial indicated a tendency toward inferior safety for TNK 0.4 mg/kg compared with 0.25 mg/kg but with a similar rate of functional outcome and reperfusion (21); currently, all ongoing randomized trials use the TNK dose of 0.25 mg/kg (7). TNK was also more often used in the treatment of wake-up strokes, which is not supported by guidelines and may have influenced the outcome (13).

Regarding efficacy, although there was substantial missing data, the absolute difference between TNK and ALT in functional independence (mRS 0–2) after 90 days (1.7% [−2.2 to 5.6%]) was within the major non-inferiority margin proposed in other studies (4). Switch to TNK in Swedish hospitals consistently led to decreased DNT in accordance with expectations and in line with most, but not all, previous studies (5, 9, 10, 22–24). Although the process time for TNK was shorter, this failed to generate an increased proportion of functional independence after 90 days, as well as a reduction in sICH and death, which previously has been associated with shorter DNT (25, 26).

Comparing with both randomized controlled trials and previous real-world data, our study included older patients who were more dependent in activities of daily living prior to stroke (4, 8–11). One may hypothesize that TNK’s increased ability to dissolve clots could implicate an increased risk in fragile patients compared with ALT, although neither these data nor earlier subgroup analyses can support such a difference between the two agents (27, 28).

This study underlines the importance of continued scrutiny of the safety of TNK in ischemic stroke. Most studies concluding non-inferiority of TNK compared to ALT focused mainly on the rate of favorable functional outcome where a wider non-inferiority margin is acceptable than in safety aspects. Less focus has been given to mortality or deterioration among those with moderate to severe disability, where deterioration is expected to have a greater impact on quality of life (29). It is therefore crucial that evaluations of new stroke treatments keep focus on safety with narrow non-inferiority margins to avoid promoting small improvements for the majority, at the cost of great deterioration in quality of life for a few. As TNK still has not proven superior to ALT in favorable functional outcome, a continued scrutiny of safety is even more essential. Although randomized controlled trials and real-world studies to date indicate comparable safety and no report has concluded inferior safety for TNK, true non-inferiority in safety outcomes with an acceptable narrow margin is yet to be proven (4, 5, 8–11).^,^ Further randomized trials are ongoing that may shed light on the safety of TNK. As the comprehensive randomized Act- and TRACE-2 trials recently published, with non-inferior functional outcome comparing TNK with ALT (5, 30), more stroke centers worldwide are expected to switch to TNK; therefore, it is also important to further survey the safety aspects of this switch in the real-world.

Being an observational register study, there are several limitations to this investigation. There could be a significant amount of selection bias at the hospital level that we cannot measure; population characteristics, indications for thrombolysis treatment, dedicated stroke competence, easy access to thrombectomy as well as advanced rehabilitation and outpatient palliative care differ between stroke centers, with TNK mostly used in specialized non-university hospitals but seldom used in university hospitals. As the data were retrospectively collected and the usage of TNK increased dramatically over time, there may also be temporal improvements or deteriorations in general stroke care that may distort the comparison between ALT and TNK. The choice of the composite endpoint of sICH and death, and a combination of TNK doses of 0.25 mg/kg and 0.4 mg/kg in the primary analysis was decided to increase the power to detect safety concerns, which is a major concern while using an off-label drug. However, this may decrease comparability with other stroke studies. Some data in the register were missing, decreasing the reliability of the mRS and NIHSS scores. We were able to control for several important confounding factors. Still, unmeasured confounding could have affected the results; for example, we had no information on NIHSS change after 24 h, time from symptom to door as well as mRS before stroke, presence of large vessel occlusion, blood sugar and blood pressure on admission. However, it is unlikely that these variables were related to the choice of treatment. The major strengths of the study were a low rate of selection bias at the patient level with physicians seldom choosing substance after examining the patient, as 97% of TNK treatments were performed after a planned institutional switch and a low rate of missing values in other variables.

## Conclusion

In this study, non-inferiority of TNK compared to ALT in safety outcome could not be proven using register data; further monitoring safety of TNK in real world data is essential. Instead, TNK was associated with increased negative outcome and decreased DNT.

## Data Availability

Because of the sensitive nature of the data collected for this study, requests to access the original data set from qualified researchers trained in human subject confidentiality protocols may be sent to Riksstroke at riksstroke@regionvasterbotten.se. The data underlying the analyses in this article will be shared on reasonable request to the corresponding author.

## References

[1] Emberson J, Lees KR, Lyden P, Blackwell L, Albers G, Bluhmki E, et al. Effect of treatment delay, age, and stroke severity on the effects of intravenous thrombolysis with alteplase for acute ischaemic stroke: a meta-analysis of individual patient data from randomised trials. Lancet. 2014;384:1929–35. doi: 10.1016/s0140-6736(14)60584-5 25106063 PMC4441266

[2] Berge E, Whiteley W, Audebert H, De Marchis GM, Fonseca AC, Padiglioni C, et al. European Stroke Organisation (ESO) guidelines on intravenous thrombolysis for acute ischaemic stroke. Eur Stroke J. 2021;6:I–LXII. doi: 10.1177/2396987321989865 PMC7995316 33817340

[3] Tanswell P, Modi N, Combs D, Danays T. Pharmacokinetics and pharmacodynamics of tenecteplase in fibrinolytic therapy of acute myocardial infarction. Clin Pharmacokinet. 2002;41:1229–45. doi: 10.2165/00003088-200241150-00001 12452736

[4] Burgos AM, Saver JL. Evidence that tenecteplase is noninferior to alteplase for acute ischemic stroke: meta-analysis of 5 randomized trials. Stroke. 2019;50:2156–62. doi: 10.1161/strokeaha.119.025080 31318627

[5] Menon BK, Buck BH, Singh N, Deschaintre Y, Almekhlafi MA, Coutts SB, et al. Intravenous tenecteplase compared with alteplase for acute ischaemic stroke in Canada (AcT): a pragmatic, multicentre, open-label, registry-linked, randomised, controlled, non-inferiority trial. Lancet. 2022;400:161–69. doi: 10.1016/S0140-6736(22)01054-6 35779553

[6] Bivard A, Huang X, Levi CR, Spratt N, Campbell BCV, Cheripelli BK, et al. Tenecteplase in ischemic stroke offers improved recanalization: analysis of 2 trials. Neurology. 2017;89:62–7. doi: 10.1212/WNL.0000000000004062 28576782

[7] Coutts SB, Berge E, Campbell BC, Muir KW, Parsons MW. Tenecteplase for the treatment of acute ischemic stroke: a review of completed and ongoing randomized controlled trials. Int J Stroke. 2018;13:885–92. doi: 10.1177/1747493018790024 30035698

[8] Katsanos AH, Psychogios K, Turc G, Sacco S, de Sousa DA, De Marchis GM, et al. Off-label use of tenecteplase for the treatment of acute ischemic stroke: a systematic review and meta-analysis. JAMA Netw Open. 2022;5:e224506. doi: 10.1001/jamanetworkopen.2022.4506 35357458 PMC8972028

[9] Gerschenfeld G, Liegey JS, Laborne FX, Yger M, Lyon V, Checkouri T, et al. Treatment times, functional outcome, and hemorrhage rates after switching to tenecteplase for stroke thrombolysis: insights from the TETRIS registry. Eur Stroke J. 2022;7:358–64. doi: 10.1177/23969873221113729 36478758 PMC9720850

[10] Zhong CS, Beharry J, Salazar D, Smith K, Withington S, Campbell BCV, et al. Routine use of tenecteplase for thrombolysis in acute ischemic stroke. Stroke. 2021;52:1087–90. doi: 10.1161/strokeaha.120.030859 33588597

[11] Tsivgoulis G, Katsanos AH, Christogiannis C, Faouzi B, Mavridis D, Dixit AK, et al. Intravenous thrombolysis with tenecteplase for the treatment of acute ischemic stroke. Ann Neurol. 2022;92(3):349–57. doi: 10.1002/ana.26445. 35713213

[12] Alamowitch S, Turc G, Palaiodimou L, Bivard A, Cameron A, De Marchis GM, et al. European Stroke Organisation (ESO) expedited recommendation on tenecteplase for acute ischaemic stroke. Eur Stroke J. 2023;8:8–54. doi: 10.1177/23969873221150022 37021186 PMC10069183

[13] Socialstyrelsen. Nationella riktlinjer för vård vid stroke. 2020-1–6545, Socialstyrelsen; 2020. Available from: https://www.socialstyrelsen.se/globalassets/sharepoint-dokument/artikelkatalog/nationella-riktlinjer/2020-1-6545.pdf [cited 15 April 2022].

[14] Riksstroke. Stroke Och TIA, Årsrapport Från Riksstroke 2020. Report, Riksstroke; 2021. Available from: https://www.riksstroke.org/wp-content/uploads/2021/12/Riksstroke_Arsrapport_2020.pdf [cited 23 March 2022].

[15] Söderholm A, Stegmayr B, Glader EL, Asplund K; Riksstroke Collaboration. Validation of Hospital performance measures of acute stroke care quality. Riksstroke, the Swedish Stroke Register. Neuroepidemiology. 2016;46:229–34. doi: 10.1159/000444679 26975057

[16] Kasner SE. Clinical interpretation and use of stroke scales. Lancet Neurol. 2006;5:603–12. doi: 10.1016/s1474-4422(06)70495-1 16781990

[17] Eriksson M, Appelros P, Norrving B, Terént A, Stegmayr B. Assessment of functional outcome in a national quality register for acute stroke: can simple self-reported items be transformed into the modified Rankin Scale? Stroke. 2007;38:1384–6. doi: 10.1161/01.STR.0000260102.97954.9c 17322093

[18] Kvistad CE, Næss H, Helleberg BH, Idicula T, Hagberg G, Nordby LM, et al. Tenecteplase versus alteplase for the management of acute ischaemic stroke in Norway (NOR-TEST 2, part A): a phase 3, randomised, open-label, blinded endpoint, non-inferiority trial. Lancet Neurol. 2022;21:511–9. doi: 10.1016/S1474-4422(22)00124-7 35525250

[19] Riksstroke. Stroke Och TIA, Årsrapport Från Riksstroke 2019. Report, Riksstroke; 2020. Available from: https://www.riksstroke.org/wp-content/uploads/2020/09/Riksstroke_A%CC%8Arsrapport-2019_slutversionWEB-1.pdf [cited 23 March 2022].

[20] Riksstroke. Stroke Och TIA, Årsrapport Från Riksstroke 2018. Report, Riksstroke; 2019. Available from: https://www.riksstroke.org/wp-content/uploads/2019/09/Riksstroke_A%CC%8Arsrapport-2018_slutversionWEB.pdf [cited 23 March 2022].

[21] Campbell BCV, Mitchell PJ, Churilov L, Yassi N, Kleinig TJ, Dowling RJ, et al. Effect of intravenous tenecteplase dose on cerebral reperfusion before thrombectomy in patients with large vessel occlusion ischemic stroke: the EXTEND-IA TNK part 2 randomized clinical trial. JAMA. 2020;323:1257–65. doi: 10.1001/jama.2020.1511 32078683 PMC7139271

[22] Warach SJ, Dula AN, Milling TJ, Miller S, Allen L, Zuck ND, et al. Prospective observational cohort study of tenecteplase versus alteplase in routine clinical practice. Stroke. 2022;53:3583–93. doi: 10.1161/STROKEAHA.122.038950 36148657

[23] Mahawish K, Gommans J, Kleinig T, Lallu B, Tyson A, Ranta A. Switching to tenecteplase for stroke thrombolysis: real-world experience and outcomes in a regional stroke network. Stroke. 2021;52:e590–3. doi: 10.1161/STROKEAHA.121.035931 34465202

[24] Hall J, Thon JM, Heslin M, Thau L, Yeager T, Siegal T, et al. Tenecteplase improves door-to-needle time in real-world acute stroke treatment. Stroke Vasc Interv Neurol. 2021;1:e000102. doi: 10.1161/SVIN.121.000102 PMC12512332 41584456

[25] Lees KR, Emberson J, Blackwell L, Bluhmki E, Davis SM, Donnan GA, et al. Effects of alteplase for acute stroke on the distribution of functional outcomes: a pooled analysis of 9 trials. Stroke. 2016;47:2373–9. doi: 10.1161/STROKEAHA.116.013644 27507856 PMC5024752

[26] Darehed D, Blom M, Glader EL, Niklasson J, Norrving B, Eriksson M. In-hospital delays in stroke thrombolysis: every minute counts. Stroke. 2020;51:2536–9. doi: 10.1161/STROKEAHA.120.029468 32586222

[27] Yogendrakumar V, Churilov L, Mitchell PJ, Kleinig TJ, Yassi N, Thijs V, et al. Safety and efficacy of tenecteplase in older patients with large vessel occlusion: a pooled analysis of the EXTEND-IA TNK trials. Neurology. 2022;98:e1292–301. doi: 10.1212/WNL.0000000000013302 35017305

[28] Thommessen B, Næss H, Logallo N, Kvistad CE, Waje-Andreassen U, Ihle-Hansen H, et al. Tenecteplase versus alteplase after acute ischemic stroke at high age. Int J Stroke Off J Int Stroke Soc. 2021;16:295–9. doi: 10.1177/1747493020938306 32631157

[29] Chaisinanunkul N, Adeoye O, Lewis RJ, Grotta JC, Broderick J, Jovin TG, et al. Adopting a patient-centered approach to primary outcome analysis of acute stroke trials by use of a utility-weighted modified Rankin scale. Stroke J Cereb Circ. 2015;46:2238–43. doi: 10.1161/STROKEAHA.114.008547 PMC4519373 26138130

[30] Wang Y, Li S, Pan Y, Li H, Parsons MW, Campbell BCV, et al. Tenecteplase versus alteplase in acute ischaemic cerebrovascular events (TRACE-2): a phase 3, multicentre, open-label, randomised controlled, non-inferiority trial. Lancet. 2023;401:645–54. doi: 10.1016/S0140-6736(22)02600-9 36774935

